# Reversed Cyclic Behavior of Carbon Nanofiber-Reinforced Concrete Shear Walls

**DOI:** 10.3390/ma17235786

**Published:** 2024-11-26

**Authors:** Liang Lu, Musaab Suliman, Wanqiu Xia

**Affiliations:** Department of Disaster Mitigation for Structures, Tongji University, 1239 Siping Road, Shanghai 200092, China; 95010@tongji.edu.cn (L.L.);

**Keywords:** carbon nanofiber concrete, reversed cyclic loading, shear wall performance, concrete ductility, finite element analysis

## Abstract

This study investigates the effects of integrating carbon nanofibers (CNF) into concrete to enhance the mechanical properties and reversed cyclic behavior of framed shear walls, addressing the need for improved seismic performance and durability. Despite the known benefits of CNF in improving concrete properties and enabling structural health monitoring, its application in framed shear walls has been limited. Through the design and testing of nineteen CNFC formulations, this research established a constitutive relationship allowing the Cyclic Softened Membrane Model (CSMM) to be applied to CNFC. Finite element analysis of shear walls under reversed cyclic loading revealed notable improvements in shear force capacity and ductility when CNF was incorporated. These findings highlight the dual role of CNFC in advancing both material performance and structural resilience, offering a significant contribution to the fields of material science and earthquake engineering.

## 1. Introduction

Carbon nanofibers and nanotubes, originally discovered by S. Iijima [1], are nanoscale fibers and tubes made of rolled-up two-dimensional carbon sheets. Carbon nanofiber (CNF) has outstanding electrical properties and unique, excellent mechanical properties, including extremely high Young’s modulus, high tensile strength, and enormous bending flexibility. These unusual mechanical properties make carbon nanotubes ideal for super strong nanofibers, thus holding great promise for synthesizing new materials [2,3].

Carbon nanofiber concrete (CNFC) is one such new synthetic material. Since the electrical resistivity variation of CNFC can indicate the state of stress and damage inside a structural specimen, and also because of its advantage in durability and consistency, CNFC has been increasingly used for online, non-destructive structural health monitoring without the need for any other sensors. This will lead to a significant reduction in the expenses associated with the upkeep and repair of infrastructure.

Carbon nanofiber concrete (CNFC) possesses not only health monitoring capabilities but also superior mechanical properties compared to conventional concrete. Research by Chen and Chung [4] revealed that the integration of low-pitch-content carbon fiber and water-reducing agents increased concrete’s flexural strength by 85%, and its compressive strength increased by 22% as a consequence of adding chemicals quickly. In addition, the ductility of the concrete was significantly enhanced. There was also a considerable reduction in electrical resistance, with a maximum decrease of 83% [5]. Hughes [6] demonstrated that incorporating carbon nanofibers into fly ash with low carbon content could dramatically augment the compressive strength of macro-defect-free (MDF) cement by 334%. Due to their tiny size, concrete infused with nano-particles displays improved characteristics compared to conventional concrete. Studies by Li et al. [7,8,9] indicated that the addition of nano-particles to concrete significantly bolstered its resistance to abrasion and flexural strain. Moreover, cement mortars mixed with nanoparticles exhibited higher compressive and flexural strengths than regular cement mortar, as evaluated after 7 and 28 days. Furthermore, due to the tunnel conductivity phenomena, the incorporation of carbon nanofibers (CNF) in concrete could potentially offer the required properties for strain measurement strain and protection against electromagnetic interference (EMI).

Amirpasha [10] conducted a comparative study on the effects of carbon nanofibers (CNF) in high-strength concrete (HSC) and ultra-high-performance concrete (UHPC). The study concluded that CNFs are more effective in UHPC than in HSC, and that steel fibers outperform PVA fibers for hybrid reinforcement with CNFs. Yanlei [11] developed embedded strain sensors based on CNF/epoxy composites. The research evaluated the sensors’ piezoresistive parameters and temperature compensation, embedded them into concrete cylinders, and monitored their compressive strains under monotonic and cyclic loadings. The study demonstrated that CNF/epoxy sensors have significant potential for the structural health monitoring of concrete structures. Hui Wang [12] assessed the flexural and compressive strengths, piezoresistivity, and de-icing performance of CNF-reinforced reactive powder concrete (CNF-RPC) with different CNF dosages. The research showed that CNF-RPC exhibits favorable mechanical properties, excellent self-sensing performance, and noticeable de-icing performance when the CNF content is close to the percolation threshold. Tengjiao [13] explored how varying amounts of CNFs influence concrete shrinkage, frost resistance, permeability, and carbonation resistance. The research found that 0.3% of CNFs is the optimal volume fraction for enhancing the durability of concrete.

Research conducted by Gao et al. [14] and Dhonde et al. [15] involved a comparative study of two types of carbon nanofiber (CNF) reinforced concrete: conventional concrete with carbon nanofibers (CNF) and self-consolidating concrete with carbon nanofibers (CNFSCC), both containing coarse aggregate. The researchers found that by integrating an optimal amount of CNF into the concrete, there was an increase in compressive strength and an improvement in the electrical properties. These enhancements are vital for monitoring strain, assessing damage, and maintaining the self-health monitoring capabilities of the concrete.

Framed shear walls, which are commonly used and essential for counteracting lateral forces, are particularly significant in structures designed to endure seismic events. This study investigates the reversed cyclic behavior of framed shear walls reinforced with carbon nanofibers by examining two varieties of such walls subjected to reversed cyclic loading. The analysis draws upon the experimental results obtained by Gao [16].

The novelty of this research lies in applying a Cyclic Softened Membrane Model (CSMM)-based finite element method to analyze CNF-reinforced shear walls, bridging the gap between material-level properties and structural-level performance. By comparing our findings with prior studies on CNF-reinforced concrete, we provide insights into how CNFs enhance both the strength and ductility of shear walls under cyclic loading, a crucial aspect not addressed in earlier research.

This study seeks to address the following research objectives: (1) to evaluate the mechanical properties of CNF-reinforced concrete, (2) to model and analyze the cyclic behavior of CNF-reinforced shear walls using finite element simulations, and (3) to assess the potential of CNFC in improving seismic performance and structural health monitoring capabilities. These findings contribute to the growing body of knowledge on nanomaterial applications in construction and provide a basis for future experimental and practical advancements.

## 2. Carbon Nano Fiber Concrete Tests

### 2.1. Materials and Mixture Proportions

Three different types of CNF (PR-19-XT-PS, PR-19-XT-PS-OX, and PR-19-XT-LHT-OX, sourced from Pyrograf Products, Cedarville, OH, USA) were used in the tests. Glenium 3200 HES, a polycarboxylate admixture, was procured from BASF Construction Chemicals, Ludwigshafen, Germany, to decrease the viscosity of the concrete [17]. Sodium dodecyl sulfate (SDS) was used to disperse the non-oxidized CNFs (such as PR-19-XT-PS) in water, sourced from Sigma-Aldrich, St. Louis, MO, USA. Antifoam 2210, an emulsion of silicone-glycol composed of polypropylene glycol and polydimethylsiloxane, was supplied by Dow Chemical Company, Midland, MI, USA, to mitigate foam generation during the mixing phase and following the addition of detergent across all three types of CNF [18].

Type I/II low-alkali cement, crushed limestone with a maximum size of 3/4″, and natural river sand with a fineness modulus of 2.71 were employed as cement, coarse aggregate, and fine aggregate, respectively.

Table 1 presents a total of nineteen distinct formulations that were developed. For each formulation, 6 cylindrical samples were cast, which included 3 cylinders measuring 8 inches in height and 4 inches in diameter, and 3 cylinders measuring 12 inches in height and 6 inches in diameter. After a period of 24 h, the specimens were removed from the molds and subsequently immersed in water at room temperature until the time of testing. Following the compression testing, select fragments of the smashed samples were chosen for further analysis using scanning electron microscopy.

The preparation of carbon nanofiber (CNF)-reinforced concrete was carefully designed to ensure uniform fiber dispersion and distribution. Three types of CNFs (PR-19-XT-PS, PR-19-XT-PS-OX, and PR-19-XT-LHT-OX) were incorporated using specific handling techniques. A polycarboxylate admixture (Glenium 3200 HES) reduced viscosity, sodium dodecyl sulfate (SDS) dispersed non-oxidized CNFs in water, and Antifoam 2210 minimized air entrapment. High-energy sonication was employed to pre-disperse CNFs before mixing to prevent agglomeration, improving the mechanical performance and durability of the material. Scanning electron microscopy (SEM) observations indicated a reasonably uniform fiber distribution with minimal clustering, though quantitative metrics for dispersion were not established. Further research is needed to quantify the effects of fiber alignment and dispersion on durability, as uniform dispersion improves load transfer and crack resistance, while clustering may create stress concentrations that compromise performance.

### 2.2. Test Results and Discussion

**Figure 1:**
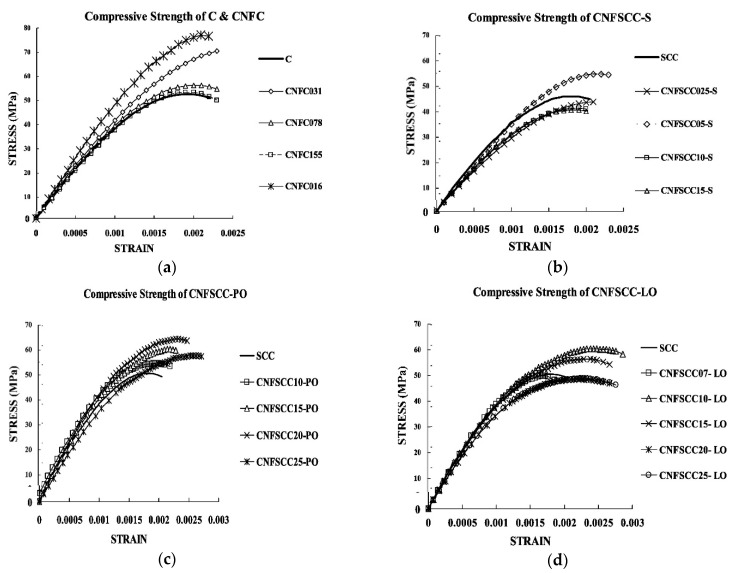
Stress vs. strain for CNFC and SCC specimens with varying concentrations of CNF.

Figure 1 depicts the stress–strain curves of CNFC and SCC specimens with different CNF concentrations. Data on the compressive strength and peak strain of 19 distinct concrete mixtures are presented in Table 2. An increase in CNF concentration results in a decrease in the ultimate strength of CNFC. However, when the CNF concentration reaches 0.16%, the compressive strength of CNFC sees a significant increase of 42.7% compared to standard concrete. This suggests that further research is needed to determine the optimal CNFC concentration in samples with a lower CNF level. Regardless of the amount of CNF added, the inclusion of CNF in concrete consistently increases its maximum deformation. The compressive strength of CNFSCC, which combines PR-19-XT-PS and SDS, shows the following variations: CNFSCC05-S is connected to SCC, which is connected to CNFSCC025-S, which is connected to CNFSCC10-S, which has a higher value than CNFSCC15-S. The concentration threshold is around 0.5%. Nevertheless, the use of SDS for assisting in the dispersion of CNF led to a notable generation of air bubbles inside the concrete, hence exerting a substantial detrimental effect on its strength. The ultimate strength of CNFSCC incorporating PR-19-XTPS-OX varies in the following sequence: CNFSCC20-PO has a higher ultimate strength than CNFSCC15-PO. The compressive strength of CNFSCC25-PO exceeds that of CNFSCC10-PO, surpassing that of SCC. Furthermore, CNFSCC25-PO shows a significant 24.4% increase in strength compared to standard SCC, especially when the CNF concentration is 2.0%. The concentration threshold is around 2.0%. The maximum compressive strength of CNFSCC containing PR-19-XT-PS-LHT-OX shows the following changes: when the concentration of cellulose nanofibers (CNF) in the self-compacting concrete (SCC) is increased to 1.0%, there is a significant 21.4% increase in the CNF concentration compared to plain SCC. The concentration threshold appears to be around 1.0%. Moreover, it is clear that regardless of the amount and type of CNF used, the peak strain of concrete consistently increases across all four variations. This observation contrasts with conventional concrete, where the peak strain decreases as the final strength increases. The preceding discussions indicate that the mechanical properties of concrete can be significantly improved by adding carbon nanofibers, provided that an appropriate concentration of CNF and mixture is used.

## 3. Tests and Simulation of Framed Shear Walls

**Figure 2:**
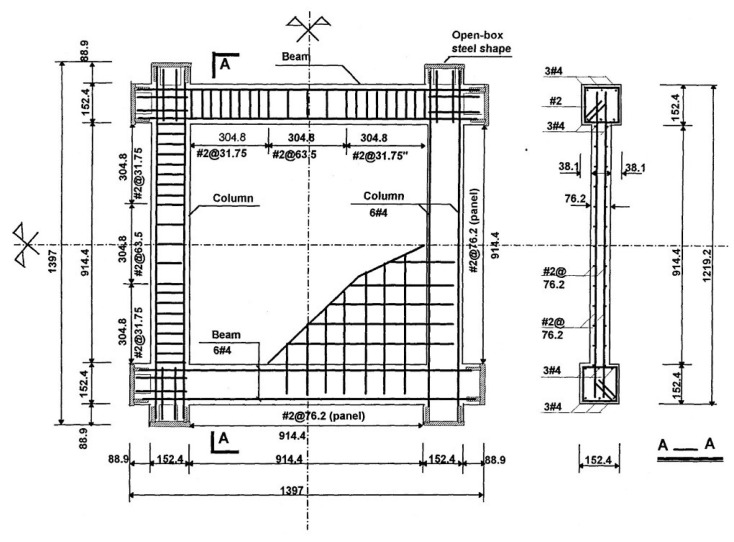
Dimensions and steel arrangement of the specimen (mm).

**Figure 3:**
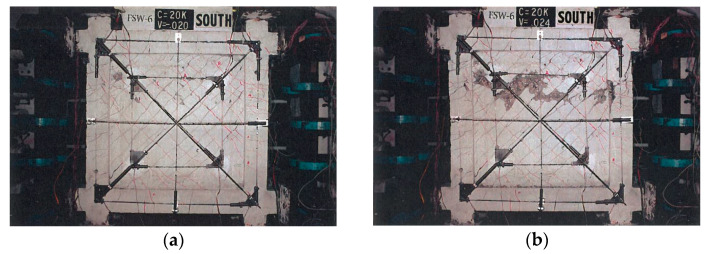
Crack pattern and failure mode of FSW-6. (**a**) Initial concrete crushing. (**b**) Final failure.

The University of Houston [16] conducted tests to assess the performance of nine 1/3-scale framed shear walls. These walls were subjected to a constant axial force at the top of each column and a cyclic load in the reverse direction at the top beam. The dimensions of the wall were 914.4 mm by 914.4 mm, with a thickness of 76.2 mm. The boundary columns displayed a cross-sectional area of 152.4 mm^2^, as depicted in Figure 2. The reinforcement features of the specimens are also illustrated. The bottom left and right corners of the specimen were supported by a hinge and a roller, respectively. Figure 3 presents the fracture pattern and failure mode of specimen FSW-6 as observed during the experiment.

### Test Methods

This section outlines the procedures employed for preparing, testing, and analyzing the carbon nanofiber-reinforced concrete (CNFC) specimens and framed shear walls subjected to reversed cyclic loading. Nineteen concrete mixtures incorporating varying concentrations of carbon nanofibers (CNFs) were designed (Table 1). For each mix, cylindrical specimens were prepared to evaluate compressive strength and peak strain. The CNFs were dispersed using a high-energy sonication process with sodium dodecyl sulfate (SDS) to ensure uniform distribution. After mixing, the fresh concrete was cast into molds, compacted, and cured in water at 25 °C for 28 days before testing. Compression tests were conducted on standard cylindrical specimens (100 mm diameter × 200 mm height) using a universal testing machine. The stress–strain behavior was recorded, and peak compressive strength and strain were determined. Three replicates were tested for each mix to ensure statistical reliability. Framed shear wall specimens were modeled based on previously tested designs from the literature. These models were analyzed using the Cyclic Softened Membrane Model (CSMM)-based finite element software. The shear walls were subjected to reversed cyclic loading under displacement-controlled conditions, simulating seismic forces. The setup included fixed boundary conditions at the base and loading points at the top to replicate realistic structural scenarios. The experimental data were processed to derive key performance metrics, including compressive strength, shear force capacity, and ductility. Finite element analysis results were compared against experimental findings from the literature to validate the simulation outcomes. Stress–strain curves, load–displacement responses, and failure patterns were analyzed to evaluate the effect of CNF incorporation.

The experimental results reveal that in the case of FSW-4, the concrete underwent rapid crushing when the steel hit its yield point, leading to a significant drop in shear force in the descending section. On the other hand, for FSW-6, the steel bars showed considerable yielding before the concrete was crushed, resulting in prolonged yield plateaus. Moreover, the ductility value of FSW-6 stands at 4.59, while that of FSW-4 is 2.78. Consequently, this study primarily investigates and assesses two common shear walls, with their respective dimensions and features detailed in Table 3. Gao [16] provides a comprehensive description of the test details and the outcomes of the experiments.

**Figure 4:**
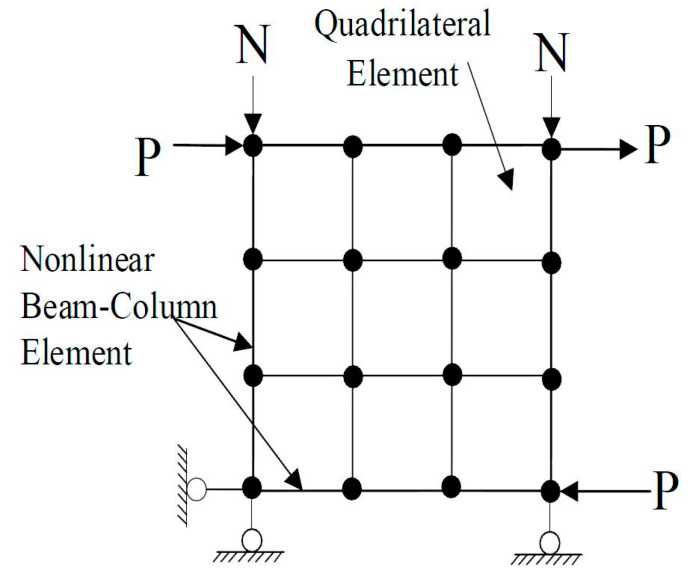
Finite element modeling of the specimen.

Finite element evaluations were performed on the two specimens. Figure 4 illustrates the specimens modeled using the finite element mesh. The wall panel consisted of nine RCPlaneStress quadrilateral parts derived from the Cyclic Softened Membrane Model (CSMM) [19,20,21]. The boundary columns and beams are represented as nonlinear beam-column elements, the pre-existing element types in OpenSees [22]. Each beam and column were partitioned into three components. The columns experienced axial loads that were delivered as vertical nodal forces, which were kept constant throughout the study.

Initially, the columns were subjected to axial loads employing load control during the study. Subsequently, the axial loads were maintained at a constant level, while a predefined displacement control technique was used to apply reverse cyclic horizontal loads. At each stage of displacement, the displacements of the nodes and the related horizontal forces were measured. Additionally, the stress and strain of the elements were continuously observed.

This study utilizes FEM simulations based on the Cyclic Softened Membrane Model (CSMM) to predict the reversed cyclic behavior of CNF-reinforced shear walls. While the simulation results align with expected trends from the literature, the absence of direct experimental validation, particularly fatigue tests on CNF-reinforced specimens, limits the definitive confirmation of these findings. Experimental tests would provide critical insights into the material behavior under cyclic loading and fatigue conditions, thereby verifying the FEM results.

**Figure 5:**
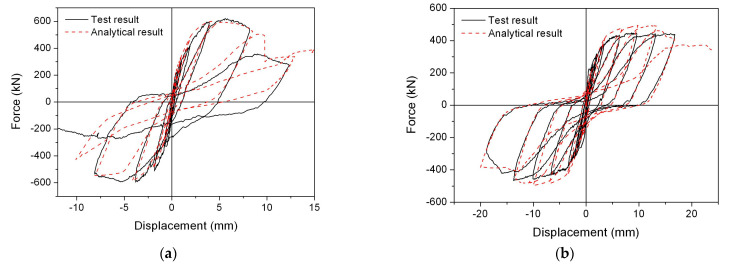
Shear force-drift displacement of specimens. (**a**) FSW-4. (**b**) FSW-6.

Figure 5 illustrates both the experimental and analytical outcomes of the shear force-drift displacement for the two shear walls. The main backbone curves, initial stiffness, yield point, peak strength, descending section, and failure characteristics all exhibited significant agreement. The hysteresis behavior offered precise quantifications of the pinching phenomena, residual displacements, ductility, and energy dissipation capacity across all specimens.

The analytical results for shear force capacity and ductility for both specimens are compared to those of the experimental outcomes, as shown in Table 4.

## 4. Simulation of Carbon Nanofiber RC FSW

The research carried out in the final section of this work indicates that the CSMM-based finite element software is highly accurate and aptly suited for performing seismic analysis of shear walls. Furthermore, it is utilized to investigate carbon nanofiber-reinforced concrete framed shear walls (CNFRC-FSW).

### 4.1. Constitutive Model for CNF

It can be noted from Figure 1 that the stress–strain curves of CNF concrete and normal concrete are similar, and the only difference is in the strength and peak strain of the concrete. This means that the constitutive model for standard concrete can also be used for CNF-reinforced concrete [23].

**Figure 6:**
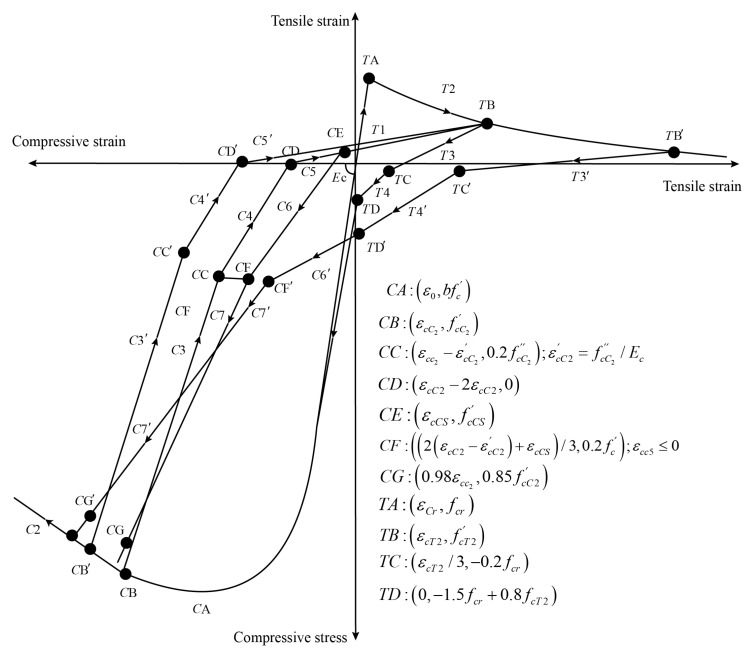
Uniaxial constitutive relationship of CNF concrete.

Based on the uniaxial constitutive law of concrete in CSMM, the cyclic uniaxial constitutive relationships of carbon nanofiber-reinforced concrete can be modified as shown in Figure 6. The characteristic of these concrete constitutive laws includes the softening effect in compression due to the tensile strain in the perpendicular direction, the softening effect in compression under reversed cyclic loading, and the opening and closing of cracks, which are taken into account during the unloading and reloading stages.

The different load stages of CNF concrete are described as follows:

(1) $$\mathrm{Stage}\;C1\;\sigma_{c}=\left(D\xi f_{cCNF}^{\prime }-f_{cT4}^{\prime }\right)\left[2\left(\frac{\bar{\varepsilon }}{\xi \varepsilon_{0CNF}}\right)-{\left(\frac{\bar{\varepsilon }}{\xi \varepsilon_{0CNF}}\right)}^{2}\right]+f_{CT4}^{\prime }\;\;0\leq |\bar{\varepsilon }|\leq \left|\xi \varepsilon_{0CNF}\right|$$

(2) $$\mathrm{Stage}\;C2\;\sigma_{c}=D\xi f_{cCNF}^{\prime }\left[1-{\left(\frac{\bar{\varepsilon }/\varepsilon_{0CNF}-1}{4/\xi -1}\right)}^{2}\right]\;\;\left|\bar{\varepsilon }|>|\xi \varepsilon_{0CNF}\right|$$

(3) $$\mathrm{Stage}\;\mathrm{UC}\;\sigma_{1}^{c}=E_{c}^{\prime }\bar{\varepsilon_{1}}+\sigma_{ci}\;\;{\bar{\varepsilon }}_{1}\leq |{\bar{\varepsilon }}_{ci}|$$

(4) $$\mathrm{Stage}\;T1\;\sigma_{c}=E_{CNF}\bar{\varepsilon }\;\;0\leq \bar{\varepsilon }\leq \varepsilon_{crCNF}$$

(5) $$\mathrm{Stage}\;T2\;\sigma_{c}=f_{crCNF}{\left(\frac{\varepsilon_{crCNF}}{\bar{\varepsilon }}\right)}^{0.4}\;\;\bar{\varepsilon }>\varepsilon_{crCNF}$$

Unloading and reloading curves are as follows:

(6) $$\sigma_{c}^{T}=\sigma_{i}^{c}+E_{cc}({\bar{\varepsilon }}_{i}-\bar{\varepsilon })$$

(7) $$E_{cc}=\frac{\sigma_{i}^{c}-\sigma_{i+1}^{c}}{{\bar{\varepsilon }}_{i}-{\bar{\varepsilon }}_{ii}}$$

where $f_{cCNF}^{\prime }$ and $\varepsilon_{0CNF}$ are the cylinder compressive strength and peak strain of CNF concrete. $f_{CT4}^{\prime }$ is the stress at point TD.

(8) $$\xi =\frac{5.8}{\sqrt{f_{cCNF}^{\prime }(MPa)}}\frac{1}{\sqrt{1+400{\bar{\varepsilon }}_{T}^{\prime }/\eta^{\prime }}}\leq 0.9$$

(9) $$D=1-0.4\frac{\varepsilon_{c}^{\prime }}{\varepsilon_{0CNF}}\leq 1.0$$

(10) $$\eta^{\prime }=\eta =\frac{\rho_{t}f_{ty}-\sigma_{t}}{\rho_{t}f_{ly}-\sigma_{l}}\leq 1.0$$

(11) $$E_{cCNF}=3875\sqrt{f_{cCNF}^{\prime }(MPa)}$$

(12) $$f_{crCNF}=0.31\sqrt{f_{cCNF}^{\prime }(MPa)}$$

(13) $$\varepsilon_{crCNF}=\varepsilon_{cr}=0.00008$$

### 4.2. Analysis of CNFRC-FSW

**Figure 7:**
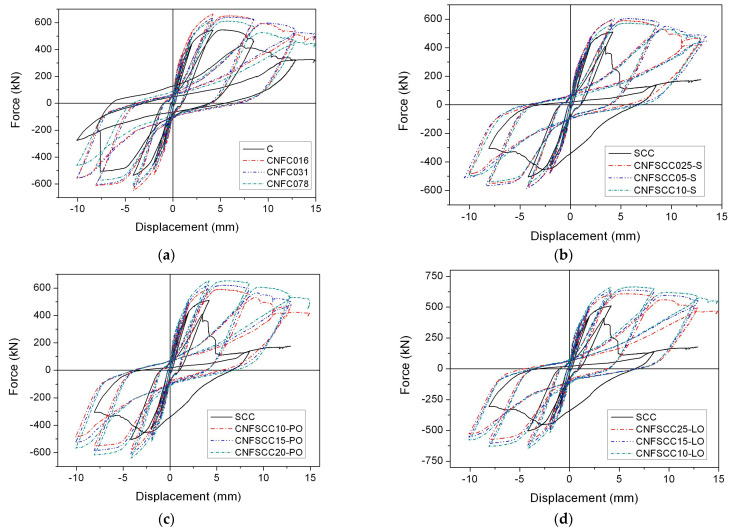
Shear force-drift displacement curves of FSW-4. (**a**) CNFC. (**b**) CNFSCC-S. (**c**) CNFSCC-PO. (**d**) CNFSCC-LO.

**Figure 8:**
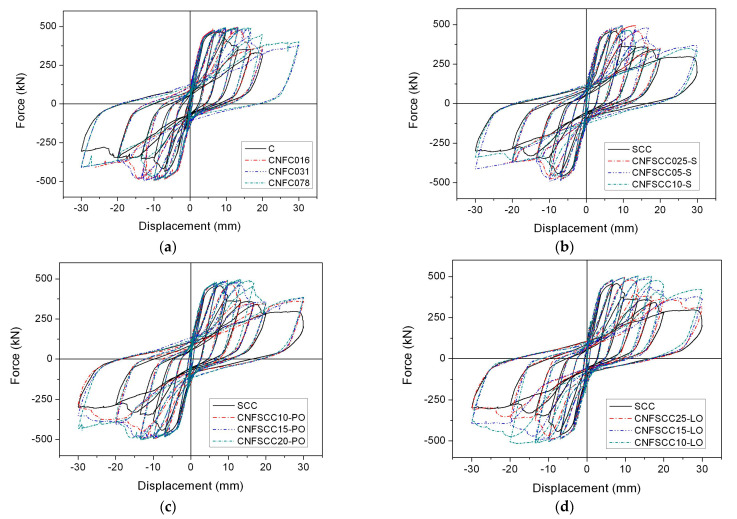
Shear force-drift displacement curves of FSW-6. (**a**) CNFC. (**b**) CNFSCC-S. (**c**) CNFSCC- PO. (**d**) CNFSCC-LO.

The carbon nanofiber-reinforced concrete framed shear walls, specifically FSW-4 and FSW-6, are modeled using four distinct types of concrete: CNFC, CNFSCC-S, CNFSCC-PO, and CNFSSC-LO. The analytical results of the shear force-drift displacement curves for these two shear walls are depicted in Figure 7 and Figure 8. Furthermore, Table 4 and Table 5 furnish information on the shear force capacity and ductility of the shear walls.

#### 4.2.1. FSW-4

Figure 6 illustrates that for SCC, the steel in FSW-4 hits its yield point just prior to the crushing of the concrete, leading to a reduction in shear force in the descending section. The ductility value of the shear wall is 2.08. In the case of FSW-4 with conventional concrete (C), even though the concrete fails before the steel, the ductility of the wall is only 2.88. Regardless of the specific type of concrete that incorporates Carbon Nanofiber (CNF), the steel element consistently reaches its yield point before the concrete experiences crushing. As a result, the ductility of the shear walls is improved. For normal concrete with CNF, the highest ductility is 5.57, an increase of 93.4%, which happens in the case of CNFC016. For the self-consolidating concrete, the highest ductility is 5.68, an increase of 173.08%, which happens in CNFSCC10-LO. It can also be found that the average increase in ductility of shear walls with CNF-reinforced normal concrete is 80.67%, compared to 102.08% in shear walls with CNFSCC, which means that reinforcing self-consolidating concrete with CNF can make shear walls more ductile. Among the three kinds of self-consolidating concrete, the shear walls with CNFSCC-LO, whose average increase in ductility is 156.41%, have higher ductility than the other two kinds of concrete.

On the other hand, the shear force capacities of shear walls reinforced with CNF are also higher than those without CNF. In the case of normal concrete, the highest increase in shear force capacity is 21.82%, which happens in the case of CNF016. For the self-consolidating concrete, the highest increase in shear force capacity is 30.37%, which happens in the case of CNFSCC10-LO. In the three kinds of SCC with different CNFs, the average increase in shear force capacity follows the order of CNFSCC-LO > CNFSCC-PO > CNFSCC-S. It is noted that the increases in shear force capacity are not as obvious as the increases in ductility of all the shear walls, which means, for FSW-4, carbon nanofiber-reinforced concrete has a better effect on the ductility than the shear force capacity.

#### 4.2.2. FSW-6

It can be found from Figure 8 and Table 6 that for the normal concrete reinforced with CNF, the increases in shear force capacity are not as obvious as those in FSW-4, and the highest increase is only 6.59%, which happens in CNFC031. However, even though FSW-6 is a ductile-designed shear wall, compared to FSW-4, the increases in its ductility are still very remarkable, and the highest increase in ductility is 73.6%, which happens in CNFC016. It is also worth noting that the highest increases in shear force capacity and ductility happened in different concrete. In contrast to normal concrete, in the case of CNFSCC-S, the average increase in the ductility of shear walls with such concrete is 9.82%, compared to 28.04% in the shear force capacity. This means that this kind of concrete affects the shear force capacity more than the ductility. In the cases of CNFSCC-PO and CNFSCC-LO, the shear force capacity and ductility of FSW-6 are improved. The highest increases in shear force capacity and ductility are 44.26% and 75.4%, respectively, and both happen in the case of CNFSCC10-LO. In general, as with FSW-4, the increase in ductility of FSW-6 is higher than the increase in shear force capacity with carbon nanofiber reinforcement.

Furthermore, according to the knowledge of engineers, even though there is no specification for ductility in the ACI code for the design of shear walls, most engineers still hope that the ductility of shear walls is higher than 4.0. From the analysis of both FSW-4 and FSW-6, it can be found that the ductility of the two shear walls without CNF is less than 4.0. However, with a reasonable concentration of CNF, the ductility values for almost all the carbon nanofiber-reinforced concrete shear walls are higher than 4.0. Therefore, CNF-reinforced concrete can be used for structure health monitoring without changing any other variables. In addition, the reversed cyclic behavior of CNFRC shear walls can also be improved remarkably. Concrete CNFSCC10-LO is suggested for use from the discussions above because it provides the greatest shear force capacity and ductility.

The reversed cyclic behavior of carbon nanofiber-reinforced concrete (CNFRC) shear walls in this study reveals shear force capacity and ductility enhancements, aligning with findings from prior research. For instance, the improvements in ductility for CNFC-reinforced normal concrete resonate with the findings of studies highlighting significant gains in compressive strength and ductility through the use of carbon nanofibers in high-performance concrete. Similarly, the performance of self-consolidating concrete (CNFSCC-LO) aligns with the results of Wang [13], emphasizing the durability and mechanical benefits of well-optimized CNF dosages. These findings collectively underscore the effectiveness of CNFRC in enhancing structural performance.

## 5. Conclusions

The mechanical characteristics of concrete, such as compressive strength and peak strain, can be significantly enhanced by incorporating carbon nanofibers (CNFs). The type and concentration of CNF are critical factors influencing these improvements. Self-consolidating concrete (SCC) demonstrates particular benefits from the inclusion of CNFs, especially oxidized variants like PR-19-XT-PS-OX and PR-19-XT-PS-LHT-OX, with optimal concentrations ranging from approximately 1.0% to 2.0%. Conventional concrete achieves its best performance with a CNF concentration of 0.16%, while SCC benefits most within a higher range. The ability to fine-tune CNF concentrations for different concrete types highlights the versatility and adaptability of this innovative material.

This study introduces a novel approach by applying the Cyclic Softened Membrane Model (CSMM) to analyze the reversed cyclic behavior of framed shear walls reinforced with CNFs. This method bridges the gap between material-level enhancements and structural performance, allowing for a comprehensive evaluation of CNF-reinforced concrete in seismic applications. The results show that CNFs significantly improve both the shear force capacity and ductility of shear walls, with these effects being more pronounced in brittle-designed shear walls. Additionally, the study confirms that CNF-reinforced SCC outperforms CNF-reinforced normal concrete in terms of reversed cyclic behavior, with PR-19-XT-LHT-OX concrete exhibiting the best overall performance.

The novelty of this research lies in demonstrating the dual role of CNFs: enhancing material properties and enabling real-time health monitoring, both of which are crucial for structural durability and resilience in seismic regions. By integrating advanced nanomaterial technology into structural design, this work paves the way for a new paradigm in earthquake-resistant construction, providing both practical solutions for improving infrastructure resilience and theoretical advancements for future research. These findings establish a strong foundation for the broader adoption of CNF-reinforced concrete in seismic design and resilient infrastructure development.

## Figures and Tables

**Table 1:** Mixture proportions of concrete.

Mix	CNF (vol.%)	Water (kg/m^3^)	Cement (kg/m^3^)	Coarse Aggregate (kg/m^3^)	Fine Aggregate (kg/m^3^)	HRWR (fl.zo./cwt)	SDS (kg/m^3^)	Antifoam (fl.zo./cwt)	Density (kg/m^3^)
C	0	188.0	572.0	1015.0	572.0	0	0	0	2347
CNFC-016	0.16	188.0	571.0	1015.0	572.0	5.0	0	0	2350
CNFC-031	0.31	188.0	570.0	1015.0	572.0	7.0	0	0	2352
CNFC-078	0.78	188.0	569.0	1015.0	572.0	16.0	0	0	2361
CNFC-155	1.55	188.0	566.0	1015.0	572.0	31.0	0	0	2375
SCC	0	191.0	480.0	901.0	1005.0	11.0	0	0	2581
CNFSCC-025-S	0.25	191.0	480.0	901.0	942.0	11.0	0.18	11.0	2525
CNFSCC-05-S	0.5	191.0	480.0	901.0	877.0	14.0	0.36	24.0	2469
CNFSCC-10-S	1	191.0	480.0	838.0	877.0	14.0	0.77	43.0	2419
CNFSCC-15-S	1.5	191.0	480.0	838.0	877.0	16.0	1.15	62.0	2433
CNFSCC-10-PO	1	191.0	478.0	902.0	1008.0	11.0	0	11.0	2600
CNFSCC-15-PO	1.5	191.0	478.0	902.0	1008.0	19.0	0	11.0	2610
CNFSCC-20-PO	2	191.0	478.0	902.0	1008.0	23.0	0	11.0	2618
CNFSCC-25-PO	2.5	191.0	478.0	902.0	1008.0	35.0	0	11.0	2629
CNFSCC-07-LO	0.7	191.0	478.0	902.0	1008.0	11.0	9	11.0	2605
CNFSCC-10-LO	1	191.0	478.0	902.0	1008.0	11.0	0	11.0	2600
CNFSCC-15-LO	1.5	191.0	478.0	902.0	1008.0	19.0	0	11.0	2610
CNFSCC-20-LO	2	191.0	478.0	902.0	1008.0	23.0	0	11.0	2618
CNFSCC-25-LO	2.5	191.0	478.0	902.0	1008.0	35.0	0	11.0	2629

Note. CNF stands for carbon nano-fiber; HRWR stands for high-range water reducer; SDS stands for sodium dodecyl sulfate; and C represents ordinary concrete. The term “***” refers to concrete that contains CNF PR-19-XT-PS. The term “CNFSCC***-S” refers to self-consolidating concrete that contains CNF PR-19-XT-PS and SDS. The term “CNFSCC***-PO” refers to self-consolidating concrete that contains CNF PR-19-XT-PS-OX. The term “CNFSCC***” refers to self-consolidating concrete. The abbreviation “LO” refers to the self-consolidation of concrete that includes CNF PR-19-XTLHT-OX.

**Table 2:** Ultimate strength and peak strain of concrete.

CNF			CNFSCC-S			CNFSCC-PO			CNFSCC-LO		
Concrete	Strength (MPa)	Peak Strain (με)	Concrete	Strength (MPa)	Peak Strain (με)	Concrete	Strength (MPa)	Peak Strain (με)	Concrete	Strength (MPa)	Peak Strain (με)
C	51.23	1817	SCC	45.51	1714	SCC	45.51	1714	SCC	45.51	1714
CNFC016	73.08	2226	CNFSCC025-S	43.44	2258	CNFSCC10-PO	47.71	2092	CNFSCC07-LO	44.75	1821
CNFC031	64.60	2737	CNFSCC05-S	51.64	2476	CNFSCC15-PO	52.33	2269	CNFSCC10-LO	55.23	2527
CNFC078	53.50	2249	CNFSCC10-S	41.16	2124	CNFSCC20-PO	56.61	2706	CNFSCC15-LO	51.16	2289
CNFC155	50.26	2108	CNFSCC15-S	39.78	2017	CNFSCC25-PO	51.57	2415	CNFSCC20-LO	43.78	2242
-	-	-	-	-	-	-	-	-	CNFSCC25-LO	44.33	2280

**Table 3:** Dimensions and properties of specimens.

Specimen	$f_{c}^{\prime }$ (MPa)	Column and Beam			Wall Panel		Vertical Load	
		Hoop Steel (mm)	Longl. Steel	Longl. Steel (%)	Panel Steel (mm)	Panel Steel (%)	P (kN)	$\frac{P}{P_{0}}Ratio$
FSW-4	49.51	ϕ6.35@63.5	6ϕ12.7	3.33	ϕ6.35@152.4	0.55	534	0.46
FSW-6	49.75	ϕ6.35@63.5	6ϕ12.7	3.33	ϕ6.35@152.4	0.55	89	0.08

**Table 4:** Shear force capacity and ductility of test and analytical results.

		FSW-4		FSW-6	
		Positive Direction	Negative Direction	Positive Direction	Negative Direction
Shear force capacity (KN)	Test	618.51	−598.34	445.28	−465.31
	Analysis	597.63	−579.44	496.39	−498.26
Ductility	Test	2.81	2.75	5.05	4.12
	Analysis	2.68	2.20	3.91	4.80

**Table 5:** Shear force capacities and ductility of FSW-4.

Concrete	Shear Force Capacity (kN)	Increment of Shear Force Capacity	Ductility	Increment of Ductility
C	545.89	0	2.88	0
CNFC016	665.00	21.82%	5.57	93.40%
CNFC031	639.59	17.16%	5.12	77.78%
CNFC078	615.02	12.66%	4.92	70.83%
Average	639.87	17.22%	5.20	80.67%
SCC	510.39	0	2.08	0
CNFSCC025-S	594.00	16.38%	4.17	100.48%
CNFSCC05-S	604.76	18.49%	4.78	129.81%
CNFSCC1-S	573.00	12.26%	3.66	75.96%
Average	590.59	15.71%	4.20	102.08%
SCC	510.39	0	2.08	0
CNFSCC10-PO	594.00	16.38%	4.89	135.10%
CNFSCC15-PO	625.00	22.46%	5.12	146.15%
CNFSCC20-PO	655.89	28.51%	5.45	162.02%
Average	624.97	22.44%	5.15	147.76%
SCC	510.39	0	2.08	0
CNFSCC10-LO	665.37	30.37%	5.68	173.08%
CNFSCC15-LO	639.48	25.53%	5.32	155.77%
CNFSCC25-LO	611.71	19.85%	5.00	140.38%
Average	638.85	25.17%	5.33	156.41%

**Table 6:** Shear force capacities and ductility of FSW-6.

Concrete	Shear Force Capacity (kN)	Increment of Shear Force capacity	Ductility	Increment of Ductility
C	467.17	0	2.88	0
CNFC016	496.21	6.22%	5.01	73.96%
CNFC031	497.97	6.59%	4.39	52.43%
CNFC078	493.73	5.69%	3.29	14.24%
Average	495.97	6.16%	4.23	46.88%
SCC	461.24	0	3.09	0
CNFSCC025-S	493.6	28.78%	3.69	19.42%
CNFSCC05-S	495.85	31.12%	3.38	9.39%
CNFSCC1-S	488.23	24.23%	3.11	0.65%
Average	590.59	28.04%	3.39	9.82%
SCC	461.24	0	3.09	0
CNFSCC10-PO	495.1	28.78%	4.13	33.66%
CNFSCC15-PO	492.9	35.50%	4.5	45.63%
CNFSCC20-PO	502.51	42.20%	5.09	64.72%
Average	624.96	35.50%	4.57	48.00%
SCC	461.24	0	3.09	0
CNFSCC10-LO	515.16	44.26%	5.42	75.40%
CNFSCC15-LO	499.61	38.64%	4.87	57.61%
CNFSCC25-LO	493.01	32.62%	3.97	28.48%
Average	638.85	38.50%	4.75	53.83%

## Data Availability

The original contributions presented in this study are included in the article. Further inquiries can be directed to the corresponding author.
